# Effect of Central Corticotropin-Releasing Factor on Hepatic Lipid Metabolism and Inflammation-Related Gene Expression in Rats

**DOI:** 10.3390/ijms22083940

**Published:** 2021-04-11

**Authors:** Yukiomi Nakade, Rena Kitano, Taeko Yamauchi, Satoshi Kimoto, Kazumasa Sakamoto, Tadahisa Inoue, Yuji Kobayashi, Tomohiko Ohashi, Yoshio Sumida, Kiyoaki Ito, Masashi Yoneda

**Affiliations:** Department of Internal Medicine, Division of Gastroenterology and Hepatology, Aichi Medical University, Nagakute, Aichi 480-1195, Japan; kitano.rena.035@mail.aichi-med-u.ac.jp (R.K.); tyamauch@aichi-med-u.ac.jp (T.Y.); kimoto.satoshi.146@mail.aichi-med-u.ac.jp (S.K.); sakamoto.kazumasa.041@mail.aichi-med-u.ac.jp (K.S.); tinoue-tag@umin.ac.jp (T.I.); kobayashi.yuuji.572@mail.aichi-med-u.ac.jp (Y.K.); oohashi.tomohiko.415@mail.aichi-med-u.ac.jp (T.O.); sumida.yoshio.500@mail.aichi-med-u.ac.jp (Y.S.); kito@aichi-med-u.ac.jp (K.I.); yoneda@aichi-med-u.ac.jp (M.Y.)

**Keywords:** corticotropin-releasing factor, SREBF1, TNFα, IL1β

## Abstract

Corticotropin-releasing factor (CRF) in the brain acts on physiological and pathophysiological modulation of the hepatobiliary system. Central CRF administration aggravates experimental acute liver injury by decreasing hepatic blood flow. Conversely, minimal evidence is available regarding the effect of centrally acting CRF on hepatic lipid metabolism and inflammation. We examined whether central CRF affects hepatic lipid metabolism and inflammation-related gene expression in rats. Male Long Evans rats were intracisternally injected with CRF (10 μg) or saline. Rats were sacrificed 2 h, 6 h, and 24 h after the CRF injection, the liver was isolated, and mRNA was extracted. Next, hepatic lipid metabolism and inflammation-related gene expression were examined. Hepatic SREBF1 (sterol regulatory element-binding transcription factor 1) mRNA levels were significantly increased 6 h and 24 h after intracisternal CRF administration when compared with those in the control group. Hepatic TNFα and IL1β mRNA levels increased significantly 6 h after intracisternal CRF administration. Hepatic sympathectomy or guanethidine treatment, not hepatic branch vagotomy or atropine treatment, inhibited central CRF-induced increase in hepatic SREBF1, TNFα and IL1β mRNA levels. These results indicated that central CRF affects hepatic de novo lipogenesis and inflammation-related gene expression through the sympathetic-noradrenergic nervous system in rats.

## 1. Introduction

Converging neuroanatomical and neuropharmacological evidence suggests that central and autonomic nervous systems participate in the regulation of hepatic functions [1,2]. The involvement of neurotransmitters mediating these effects in the central nervous system remains poorly understood. Neuropeptides are recognized as neurotransmitters in the central and peripheral nervous systems [3,4]. Notably, central acting neuropeptides regulate a variety of physiological functions. In particular, the effect of corticotropin-releasing factor (CRF) in the brain on the physiological and pathophysiological regulation of the gastrointestinal tract has been reported [5]. With regard to the gastrointestinal tract, central injection of CRF inhibited gastric motility and enhanced colonic motility through the parasympathetic vagal dependent cholinergic pathway in rats [6].

With regard to the hepatobiliary system, the autonomic nervous system affects hepatic metabolism and hemodynamics [1,7]. Electrical stimulation of the hypothalamus and continuous activation of sympathetic nerve enhance liver injury in rats [8]. There is ample evidence that the liver receives abundant innervation [9], and the autonomic nervous system influences hepatic microcirculation in rats [10]. Previously, we have revealed that central CRF administration aggravates experimental acute liver injury by decreasing hepatic blood flow through the sympathetic-noradrenergic pathway in rats [11,12].

As a consequence of the widespread Westernization of dietary patterns, the prevalence of non-alcoholic fatty liver disease (NAFLD) has reached 24% of the global population [13]. NAFLD is considered a hepatic manifestation of metabolic syndrome, which is a cluster of cardiovascular risk factors [14,15]. Indeed, the most common cause of death in NAFLD is cardiovascular disease [15]. Although the pathogenesis of non-alcoholic steatohepatitis (NASH) remains unclear, many parallel hits derived from insulin resistance, obesity with adipocyte proliferation, and gut flora may contribute to the development of NAFLD [16]. Furthermore, hepatic lipogenesis and inflammation are reportedly expedited in patients with NAFLD [17]. 

Concerns have been raised regarding the fidelity of hepatic fat metabolism, FFA influx, hepatic de novo lipogenesis, β-oxidation, and very low-density lipoprotein (VLDL) excretion in the NAFLD model [18,19]. Apolipoprotein B (*ApoB*) and microsomal triglyceride transfer protein (*MTTP*) are proteins related to the production of VLDL and excretion of triglyceride (TG) from hepatocytes [20], and sterol regulatory element-binding transcription factor 1 (*SREBF1*) which is related hepatic de novo lipogenesis, and acyl-coenzyme A oxidase 1 (*ACOX1*) which is related hepatic β-oxidation were assessed as hepatic lipid metabolism [18]. The expression of the macrophage surface marker *CD68*, inflammation related genes, *TNF*α*, IL1β,* and transforming growth factor (*TGF)-β* mRNA levels were assessed as hepatic inflammation in the NAFLD model [21]. 

Inter-organ communication through the autonomic nervous system is an essential regulator of metabolism [22]. In the brain-liver axis, neuropeptide Y (NPY) has demonstrated profound effects on body-energy homeostasis, including regulation of feeding behavior [23] and lipoprotein lipase activity [24]. Centrally acting NPY stimulates VLDL-TG production through the sympathetic nervous system [25]. Like NPY, CRF acts on the hypothalamus in the brain to affect feeding behavior [26]; however, little is known regarding the effect of centrally acting CRF on hepatic lipid metabolism and inflammation, which are found to be increased in NAFLD. 

In the present study, we examined whether central CRF affects hepatic lipid metabolism and inflammation-related gene expression in rats. We demonstrated that central CRF affected hepatic de novo lipogenesis and inflammation-related gene expression through the sympathetic-noradrenergic nervous system in rats. 

## 2. Results

### 2.1. Effect of Intracisternal CRF Injection on Hepatic Lipid Metabolism and Inflammation-Related Gene Expression

**Figure 1:**
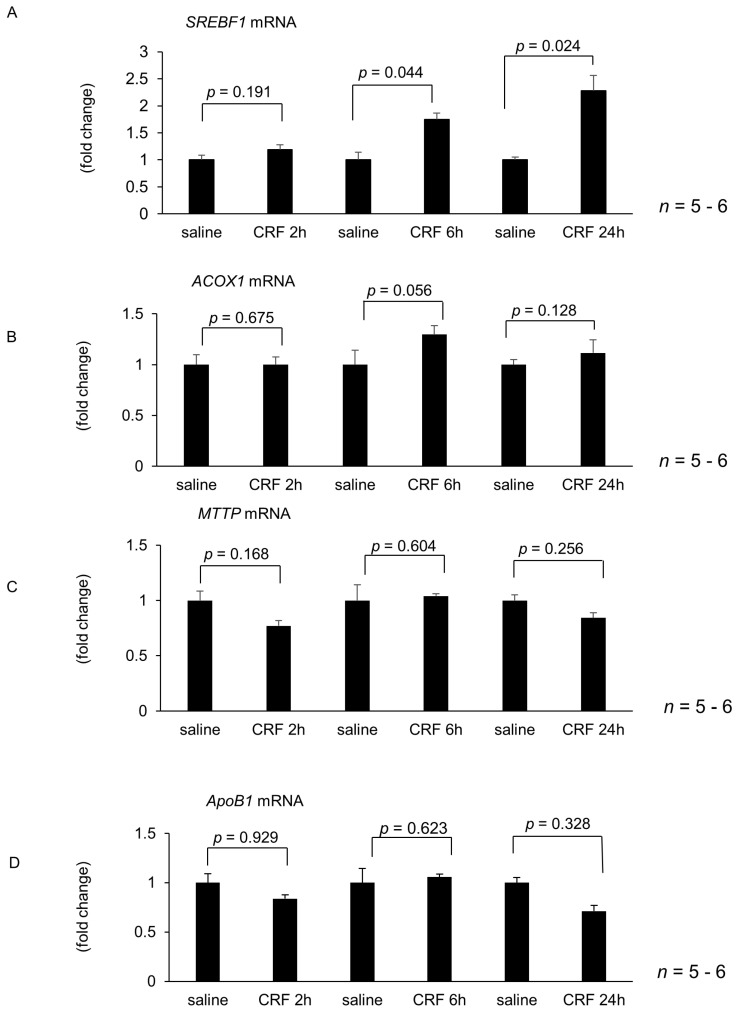

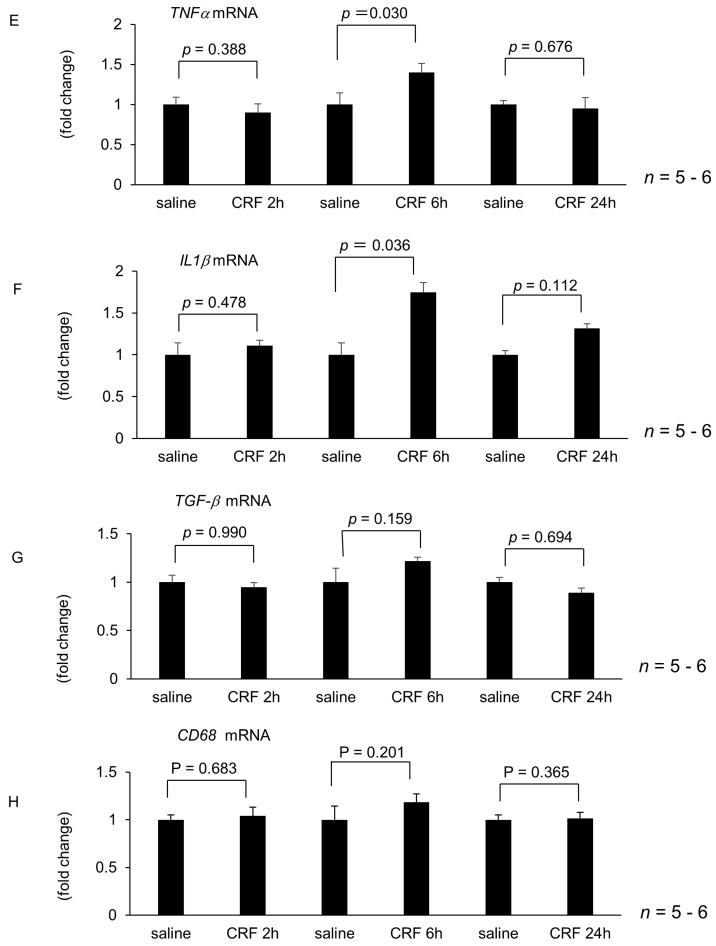
Effect of intracisternal injection of corticotropin-releasing factor (CRF) on hepatic gene expression during experimental period. Hepatic lipid metabolism and inflammation-related gene expression 2 h, 6 h, and 24 h after intracisternal CRF administration. The number of each group in rats was six. Relative mRNA expressions of *SREBF1* (**A**), *ACOX1* (**B**), *MTTP* (**C**), *ApoB1* (**D**), *TNFα* (**E**), *IL1β* (**F**), *TGFβ* (**G**), and *CD68* (**H**) were evaluated and compared with respective saline control. Statistical analysis was performed using Student’s *t*-test, and data are expressed as means ± standard error (SE). CRF, corticotropin-releasing factor; SREBF1, sterol regulatory element-binding transcription factor 1; ACOX1, acyl-coenzyme A oxidase; MTTP, microsomal triglyceride transfer protein; ApoB1, apolipoprotein B1; TGFβ, transforming growth factor-β.

Hepatic lipid metabolism and inflammation-related gene expression were unaltered 2 h after intracisternal CRF injection (Figure 1A–H). However, 6 h and 24 h after intracisternal CRF administration, hepatic *SREBF1* mRNA levels increased significantly when compared with those observed in the control (Figure 1A). In contrast, hepatic *ACOX1*, *MTTP*, and *ApoB1* mRNA expressions did not significantly change at 6 h and 24 h after intracisternal CRF administration (Figure 1B,H). Hepatic *TNFα* and *IL1β* mRNA levels significantly increased 6 h after intracisternal CRF injection (Figure 1E,F). Conversely, *TGFβ* and *CD68* mRNA levels did not increase 6 h and 24 h after intracisternal CRF administration.

Additionally, 24 h after intracisternal CRF injection, serum corticosterone levels were unaltered when compared with those of the respective control during the experimental period (Table 1). 24 h after intracisternal CRF injection, serum TG levels significantly increased when compared with those of the respective control (Table 2).

### 2.2. Effect of Central CRF Antagonist on CRF-Induced Modulation of Hepatic Lipid Metabolism and Inflammation-Related Gene Expression

**Figure 2:**
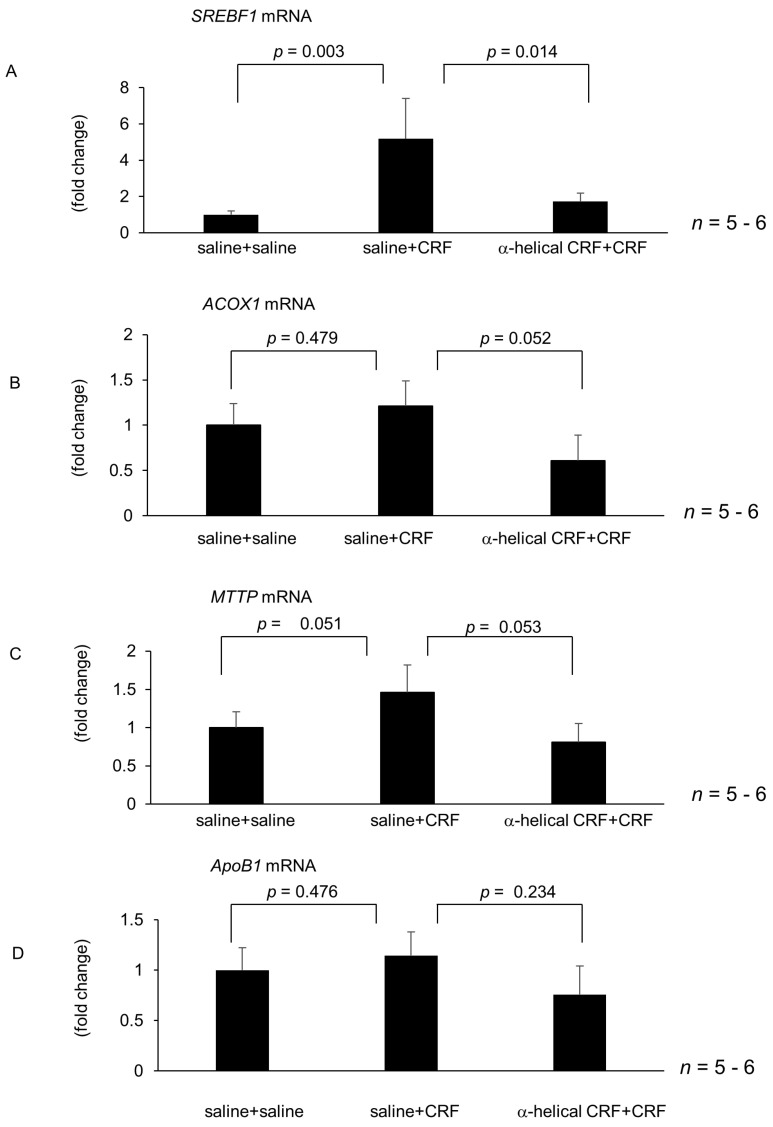

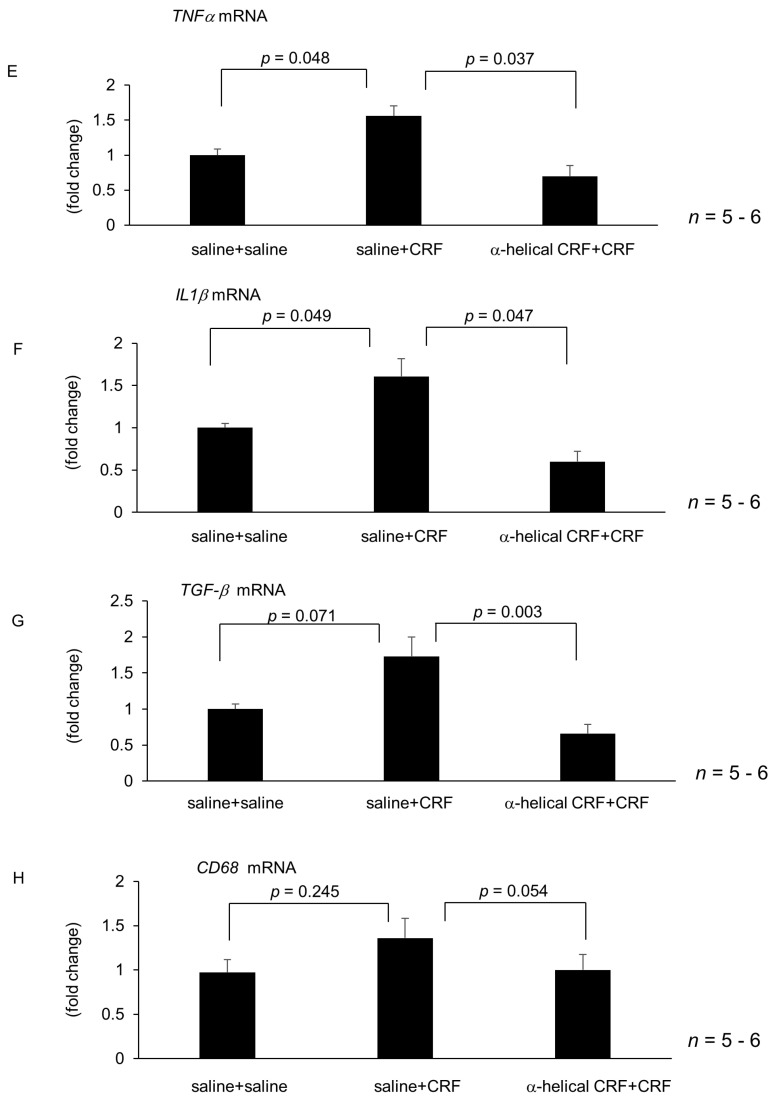
Effect of intracisternal injection of α-helical CRF on CRF-induced modulation of hepatic gene expression 6 h after intracisternal CRF administration. The number of each group in rats was six. Hepatic lipid metabolism and inflammation-related gene expression 6 h after intracisternal CRF administration. Relative mRNA expressions of *SREBF1* (**A**), *ACOX1* (**B**), *MTTP* (**C**), *ApoB1* (**D**), *TNFα* (**E**), *IL1β* (**F**), *TGFβ* (**G**), and *CD68* (**H**) were evaluated and compared with respective saline control. Statistical analysis was performed using ANOVA, and data are expressed as means ± standard error (SE).

According to the effect of central CRF on hepatic gene expressions, *SREBF1*, *TNFα* and *IL1β* mRNA levels were significantly increased at 6 h after CRF injection, following examinations were investigated at 6 h after central CRF injection. mRNA levels of hepatic SREBF1 were significantly increased 6 h after intracisternal CRF injection (Figure 2A). Intracisternal injection of α-helical CRF (9–41) inhibited CRF-induced augmentation of hepatic *SREBF1* mRNA levels 6 h after CRF injection (Figure 2A). Conversely, intracisternal injection of α-helical CRF (9–41) did not alter hepatic *ACOX1*, *MTTP*, and *ApoB1* mRNA levels induced by intracisternal CRF injection (Figure 2B–D). mRNA levels of hepatic *TNFα* and *IL1β* significantly increased 6 h after intracisternal CRF administration (Figure 2E,F). Intracisternal injection of α-helical CRF (9–41) inhibited CRF-induced augmentation of hepatic *TNFα* and *IL1β* mRNA levels 6 h after CRF injection (Figure 2E,F). In contrast, α-helical CRF (9–41) significantly inhibited CRF-induced augmentation of *TGFβ* mRNA levels 6 h after CRF injection, but did not modify *CD68* mRNA levels induced by intracisternal CRF injection (Figure 2G,H). 

### 2.3. Effect of Intravenous CRF Injection on Hepatic Lipid Metabolism and Inflammation-Related Gene Expression

**Figure 3:**
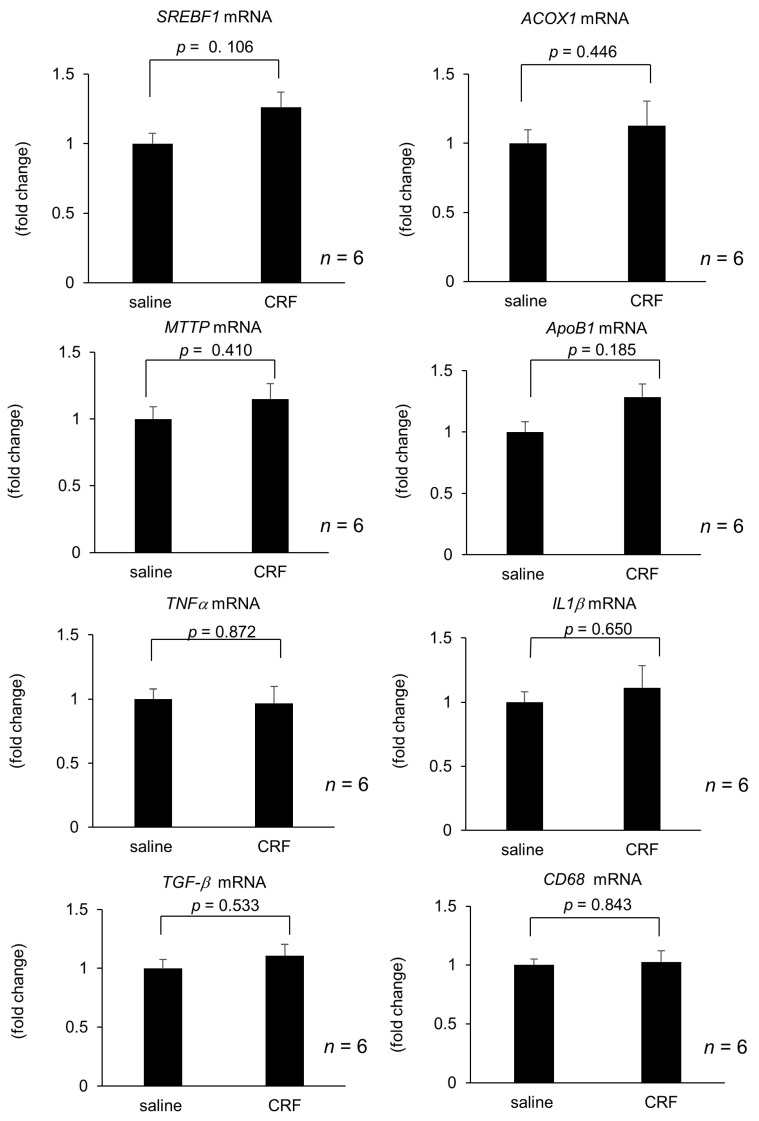
Effect of intravenous injection of CRF on hepatic gene expression during experimental period. The number of each group in rats was six. Hepatic lipid metabolism and inflammation-related gene expression 6 h after intravenous CRF administration. Relative mRNA expressions of *SREBF1, ACOX1, MTTP, ApoB1, TNFα, IL1β, TGFβ*, and *CD68* were evaluated and compared with respective saline control. Statistical analysis was performed using Student’s *t*-test, and data are expressed as means ± standard error (SE).

Hepatic *SREBF1, ACOX1, MTTP, ApoB, TNFα, IL1β, TGFβ*, and *CD68* mRNA levels were unaltered 6 h after intravenous CRF injection when compared with those observed following saline administration (Figure 3).

### 2.4. Effect of Atropine, Guanethidine, Hepatic Branch Vagotomy, and Hepatic Plexus Denervation on Intracisternal CRF-Medicated Hepatic Gene Expressions

**Figure 4:**
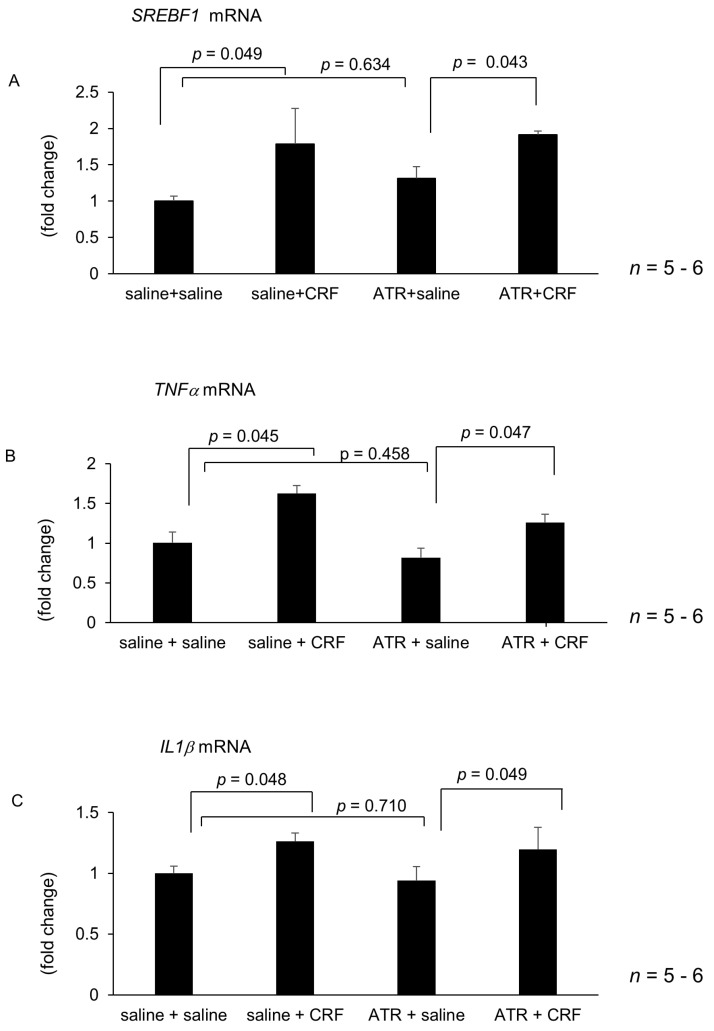

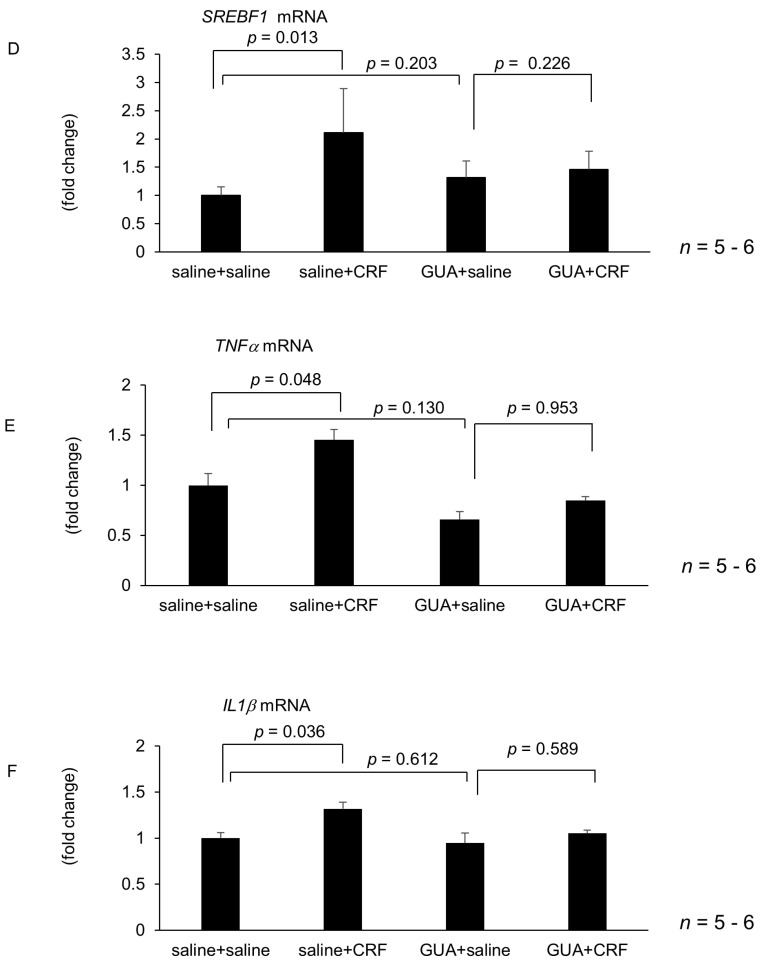
Effect of atropine (ATR) or guanethidine (GUA) on intracisternal CRF-mediated hepatic gene expression. The number of each group in rats was six. Hepatic lipid metabolism and inflammation-related gene expression 6 h after intracisternal CRF administration. Relative mRNA expressions of *SREBF1*, *TNFα*, and *IL1β* mediated by ATR (**A**–**C**) were evaluated and compared with respective saline control. Relative mRNA expressions of *SREBF1*, *TNFα*, and *IL1β* mediated by GUA (**D**–**F**) were evaluated and compared with respective saline control. Statistical analysis was performed using ANOVA, and data are expressed as means ± standard error (SE). ATR, atropine; GUA, guanethidine.

**Figure 5:**
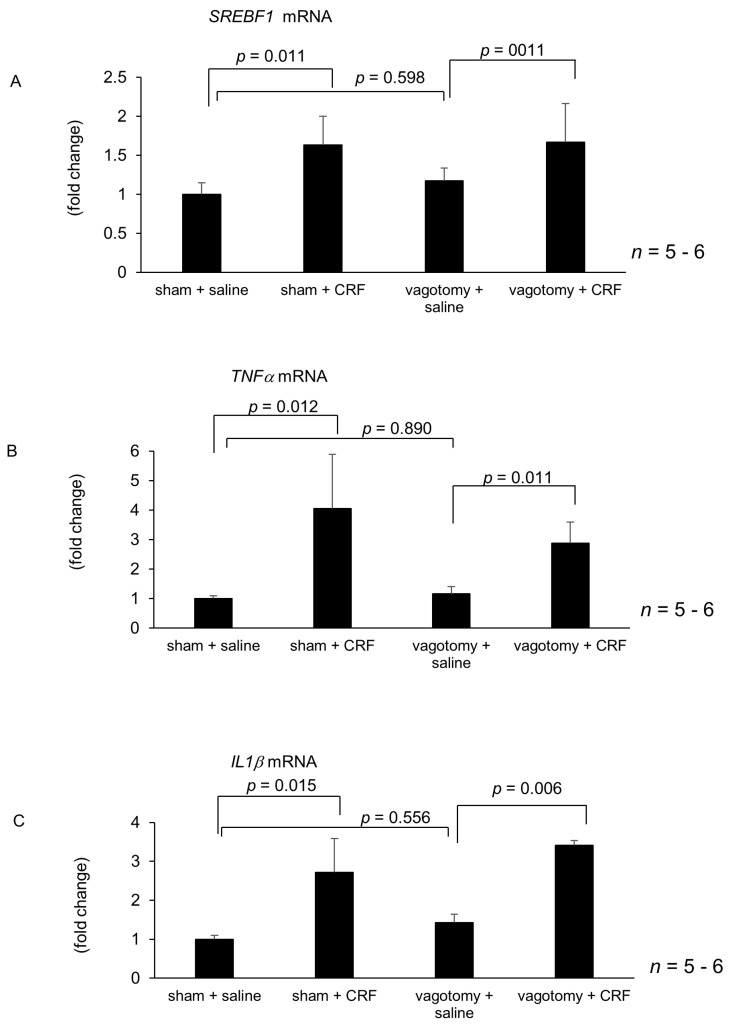

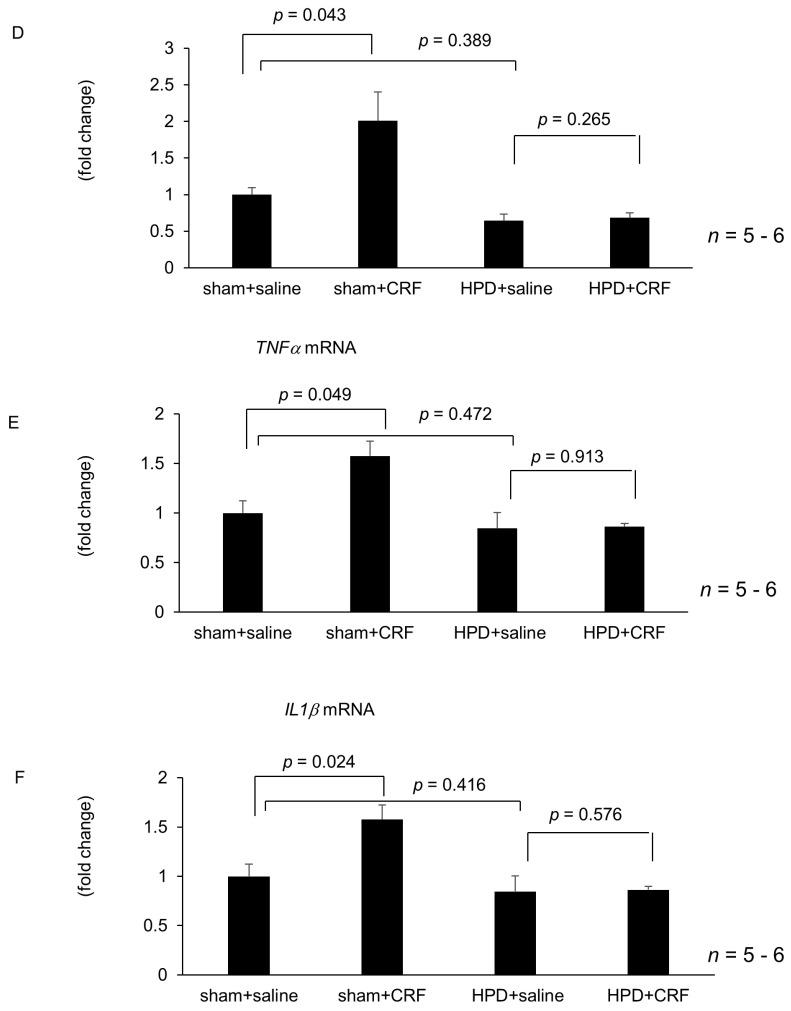
Effect of hepatic branch vagotomy or hepatic plexus denervation (HPD) on intracisternal CRF-mediated hepatic gene expression. The number of each group in rats was six. Hepatic lipid metabolism and inflammation-related gene expression 6 h after intracisternal CRF administration. Relative mRNA expressions of *SREBF1*, *TNFα*, and *IL1β* mediated by vagotomy (**A**–**C**) were evaluated and compared with respective saline control. Relative mRNA expressions of *SREBF1*, *TNFα*, and *IL1β* mediated by HPD (**D**–**F**) were evaluated and compared with respective saline control. Statistical analysis was performed using ANOVA, and data are expressed as means ± standard error (SE). HPD, hepatic plexus denervation.

Treatment with atropine and guanethidine alone failed to alter hepatic *SREBF1*, *TNFα* and *IL1β* mRNA levels 6 h after intracisternal saline injection (Figure 4A–F). Intracisternally administered CRF significantly increased mRNA levels of hepatic *SREBF1*, *TNFα* and *IL1β* following atropine treatment (Figure 4A–F). In contrast, guanethidine inhibited the intracisternal CRF-induced augmentation of hepatic *SREBF1*, *TNFα* and *IL1β* mRNA levels (Figure 4D–F). Hepatic branch vagotomy and hepatic plexus denervation alone did not alter hepatic *SREBF1, TNFα* and *IL1β* mRNA levels (Figure 5A–F). Intracisternal CRF administration significantly increased hepatic *SREBF1, TNFα* and *IL1β* mRNA levels following hepatic branch vagotomy (Figure 5A–C). Conversely, hepatic plexus denervation inhibited the central CRF-induced increase in hepatic *SREBF1, TNFα* and *IL1β* mRNA levels (Figure 5D–F).

## 3. Discussion

In the present study, we showed that intracisternally administered CRF augmented hepatic SREBF1, TNFα and IL1β mRNA levels. The augmentation of CRF-induced hepatic SREBF1, TNFα and IL1β mRNA levels was observed 6 h after CRF administration. In contrast, when injected intravenously at the same dose effective when administered intracisternally, CRF did not influence hepatic gene expression. Furthermore, we revealed that a central CRF antagonist injection abolished the CRF-induced augmentation of hepatic SREBF1, TNFα and IL1β mRNA levels. These results indicate that CRF injected into the cisterna magna acts at the central nervous system to increase hepatic SREBF1, TNFα and IL1β mRNA levels, although not through leakage into the peripheral circulation.

In this study, we further investigated pathways through which the central administration of CRF increased hepatic SREBF1, TNFα and IL1β mRNA levels. Previous reports have indicated that central CRF affects peripheral organs in part through the autonomic nervous system [6]. Regarding the digestive system, central CRF inhibits gastric secretion and motility and exocrine secretion of the pancreas through the sympathetic-noradrenergic nervous system, and the central CRF receptor antagonist partially reverses these effects [27,28]. In the present study, the effect of CRF was abolished by denervation of the hepatic plexus with phenol or guanethidine pretreatment, whereas hepatic branch vagotomy or atropine methyl nitrate treatment demonstrated no effect. Treatment of the hepatic plexus with phenol is known to predominantly denervate the hepatic sympathetic nerve, and guanethidine is an antihypertensive compound that competes with noradrenaline for transport into presynaptic terminals of adrenergic neurons via the norepinephrine transporter [1,29]. In the present study, chemical sympathectomy using 85% phenol or noradrenaline antagonist guanethidine alone did not modify hepatic SREBF1, TNFα and IL1β mRNA levels, indicating that sympathetic and noradrenergic nerve tone may not play a role in modulating hepatic gene expressions. Based on these findings, it can be suggested that CRF in the brain may play a role in sympathetic tone activation. These findings, for the first time, revealed that central CRF affects hepatic lipid metabolism and inflammation-related gene expression.

We showed that SREBP1 mRNA levels when saline was injected prior to CRF was larger than those in the first experiments. The exact reason why this phenomenon occurred remains to be elucidated. Saline injection that was administered before CRF might boost the effect of CRF.

The pathophysiological effect of stressors and the autonomic nervous system on the liver has been reported. Some stressors or enhancement of the sympathetic nervous activity are known to exacerbate experimental liver injury [8,30,31,32]. Reportedly, some physiological stressors increase CRF mRNA expression and CRF immunoreactivity in the hypothalamus and amygdala [33,34], and endogenous CRF regulates stress-induced alteration of gastrointestinal functions or chemical-induced liver injury through the autonomic nervous system [27,35,36]. Furthermore, obesity-induced hepatic steatosis is associated with robust hepatic sympathetic overactivity [37]. We demonstrated that CRF acts in the central nervous system and increases SREBF1, TNFα and IL1β mRNA levels in the liver. It may be interesting to investigate the role of endogenous CRF in high fat diet-induced hepatic steatosis and inflammation using a potent CRF antibody or antagonist in an experimental NAFLD model.

CRF nerve fibers and receptors are widely distributed in the central nervous system [38], and the site of CRF action needs to be investigated as microinjection of CRF into specific brain nuclei was not performed. In the present study, we injected CRF agonist and antagonist into the cisterna magna, which is close to the medulla. Therefore, it can be suggested that the site of CRF antagonist action is near the medulla as CRF nerve terminals and receptors are located in the nuclei in this area [38]. CRF mediates its actions through activation of specific seven-transmembrane domain receptors, which are coupled to a guanine nucleotide stimulatory factor signaling protein, resulting in increased intracellular cAMP levels [39]. To date, two CRF receptor subtypes, designated CRF1 and CRF2, have been identified through molecular cloning from distinct genes in the rats and humans [39,40]. CRF1 is located on brain neurons, whereas CRF2 is found in nonneuronal brain tissues and in the periphery [40,41]. The determination of CRF subtypes involved in the regulation of hepatic lipid metabolism and inflammation-related genes expression is warranted.

We revealed that central CRF increased serum TG levels at 24h after administration, indicating that VLDL-TG production might be increased by sympathetic nerve activity. Stafford et al. have reported that the central infusion of NPY increases serum VLDL-TG levels [42]; however, VLDL-TG excretion related genes expressions were not changed, and the underlying mechanism remains unclear. Reportedly, this mechanism is part of the physiological response during fasting when lipids become the main energy source. First, NPY neurons in the arcuate nucleus (ARC) of the hypothalamus are activated in response to fasting, and the extracellular availability of NPY in the paraventricular nucleus (PVN) is increased [43,44,45]. Second, Viñuela and Larsen have shown that intracerebroventricular administration of NPY activates neurons in the PVN projecting to the sympathetic preganglionic neurons [46]. Third, it has been revealed that preautonomic neurons in the PVN are anatomically connected to the liver [47,48]. These pharmacological and anatomical data support the concept that NPY neurons in the ARC communicate with peripheral metabolic organs via the sympathetic nervous system. Accordingly, the central administration of CRF in the PVN, which is anatomically connected to the liver, results in the augmentation of sympathetic tone. Although hepatic SREBF1, TNFα and IL1β mRNA levels increased, responsive site in the liver remains to be elucidated. It has been shown that liver shows a rich hepatocyte innervation, and catecholaminergic nerves contact Kupffer cells, endothelial lining cells, or stellate cells [1]. Thus, CRF might affect hepatocyte or Kupffer cell which potent increase SREBF1 or TNFα and IL1β thought the sympathetic nerve.

In conclusion, central CRF acts in the central nervous system to augment hepatic de novo lipogenesis and inflammation-related gene expression through the sympathetic-noradrenergic nervous system in rats. These results indicate that brain-liver interaction may regulate hepatic lipid metabolism and inflammation.

## 4. Materials and Methods

### 4.1. Substances and Treatments

The following substances were used during experimentation: rat CRF (Peptide Institute, Osaka, Japan) and a CRF receptor antagonist, α-helical CRF (9–41) (Sigma, St. Louis, MO, USA); CRF and α-helical CRF (9–41) were dissolved in 0.9% saline (pH 7.4) before the experiment and intracisternally injected (10 μL) using a 50-μL Hamilton microsyringe (Hamilton, Reno, NV, USA). Guanethidine (5.0 mg/kg intraperitoneal [ip]) and atropine sulfate (50 μg/kg ip) (Sigma, St. Louis, MO, USA) were dissolved in 0.9% saline before the experiment.

### 4.2. Animal Model and Experimental Design

Seven-week-old male *Long Evans* rats were purchased from Charles River (Yokohama, Japan) and were maintained under controlled temperature (22–24 °C) and illumination (12-h light cycle starting at 6:00 AM) for at least 7 days before experiments. Animals were maintained on laboratory chow and water during the acclimatization period, and protocols describing the use of rats were approved by the Institutional Animal Care and Use Committee of Aichi Medical University, in accordance with the National Institutes of Health “Guide for the Care and Use of Laboratory Animals.” Before the experiment, rats were deprived of food for 12 h but given free access to water up to the beginning of the study. Rats were anesthetized using isoflulene (4%), mounted on ear bars of a stereotaxic apparatus (model 900, David Kopf Instruments, Tujunga, CA, USA), and intracisternally injected with CRF (10 μg) or saline. The CRF dose was determined based on a previous report [11]. The presence of cerebrospinal fluid in the syringe on aspiration before injection verified the accuracy of needle placement into the cisterna magna. Thirty-six rats were divided into six groups and maintained in individual cages and were sacrificed 2 h, 6 h, and 24 h after CRF saline injection, respectively. A separate control group was sampled at each time points. Twelve rats were divided into two groups and maintained in individual cages. We also administered a CRF intravenously at a same dose which were applied intracisternaly, and then rats were sacrificed. After CRF or saline injection, rats were not given food but free access water until rats were sacrificed. Additionally, a liver sample was obtained from the left hepatic lobe; the sample was frozen in liquid nitrogen and stored at −80 °C. Blood samples were collected from the left ventricle and centrifuged, and the serum was stored at −80 °C.

### 4.3. Serum Biochemical Measurements

Serum TG levels were measured using a triglyceride detection kit (Wako, Osaka, Japan). Serum corticosterone levels were measured using an ELISA kit (Cayman Chemical, Ann Arbor, MI, USA).

### 4.4. Real-Time Polymerase Chain Reaction (PCR)

Frozen liver specimens were crushed in TRIzol reagent (Life Technologies, Tokyo, Japan) and RNA extraction was performed using the RNeasy Mini Kit (Qiagen, Tokyo, Japan). The isolated RNA was resuspended in 40 μL of RNase-free water and quantified by spectrophotometry (optical density [OD] 260 and low-mass gel electrophoresis [Invitrogen, Tokyo, Japan]). The extracted total RNA was reverse transcribed to cDNA using the High-Capacity cDNA Reverse Transcriptional Kit (Applied Biosystems, Foster City, CA, USA) according to the manufacturer’s instructions. Real-time quantitative PCR analysis of RNA was performed with the ABI Step One Sequence Detection System (Applied Biosystems), using TaqMan Gene Expression Assays and TaqMan Universal PCR Master Mix (Applied Biosystems), according to the manufacturer’s instructions. The detailed protocol for TaqMan PCR was determined based on a previous study [49]. 

Expressions of apolipoprotein B (*ApoB*), microsomal triglyceride transfer protein (*MTTP*), sterol regulatory element-binding transcription factor 1 (*SREBF1*), and acyl-coenzyme A oxidase 1 (*ACOX1*) were assessed as these genes are involved in hepatic lipid metabolism. To examine the inflammatory response, we assessed the expression of the macrophage surface marker *CD68*, *TNF*α*, IL1β,* and transforming growth factor (*TGF)-β* mRNA levels. (Taqman genes ID: [*ACOX1*], Rn01460628_m1; [*ApoB*], Rn01499054_m1; [*CD68*], Rn01495634_g1; [*IL1β*], Rn00580432_m1; [*SREBF1*], Rn01495769_ m1; [*MTTP*], Rn01522963_m1; [*TNFα*], Rn00562055_m1; [*TGF-β*], Rn00572010_m1).

### 4.5. Effect of Central CRF Antagonist on CRF-Induced Modulation of Hepatic Gene Expressions

To investigate whether CRF-induced modulation of hepatic gene expression is mediated by central or peripheral CRF receptors, α-helical CRF (9–41), a novel CRF antagonist, was administered intracisternally 30 min before CRF injection. Then, rats were sacrificed 6 h after CRF injection. A liver sample was obtained from the left hepatic lobe; the sample was frozen in liquid nitrogen and stored at −80 °C. Blood samples were collected from the left ventricle and centrifuged, and the serum was stored at −80 °C.

### 4.6. Effect of Atropine, Guanethidine, Hepatic BRANCH Vagotomy and Hepatic Plexus Denervation on CRF-Induced Modulation of Gene Expression

Atropine methyl nitrate (0.15 mg/kg) or guanethidine (5 mg/kg) were dissolved in saline and intraperitoneally administered (1.0 mL/kg) 30 min before CRF injection. Hepatic branch vagotomy or sham operation was performed under isoflulene anesthesia 7 days before the peptide injection. Hepatic branch vagotomy was achieved under a dissection microscope using a selective section of the hepatic branch of the vagus nerve branching off from the anterior vagal trunk, a few millimeters proximal to the cardia [50]. Hepatic plexus denervation or sham operation was performed under isoflulene anesthesia 7 days before the peptide injection, according to the method described by Lautt [1]. Denervation of the hepatic plexus (anterior plexus and posterior plexus) was achieved rapidly (<20 min), by applying phenol (85%) to the region where the hepatic artery and the portal vein run in close apposition.

### 4.7. Statistical Analysis

Data are expressed as mean ± standard error (SE). Comparison between two independent groups was performed by Student’s *t* test. Multiple comparison was calculated by an analysis of variance (ANOVA) followed by Bonferroni post hoc test. A P value of less than 0.05 was considered statistically significant. 

## Figures and Tables

**Table 1:** The changes of serum corticosterone levels in each time points in rats.

Group	*n*	0 h	2 h	6 h	24 h
CRF	6	0.42 ± 0.02	0.69 ± 0.03	0.61 ± 0.05	0.60 ± 0.05
saline	6	0.38 ± 0.05	0.59 ± 0.09	0.71 ± 0.07	0.50 ± 0.06

Serum corticosterone levels (ng/dL) were measured following intracisternal injection of CRF for 2 h, 6 h and 24 h. Data are expressed as means ± SE; ng/mL, Statistical comparison were made using Student’s *t*-test.

**Table 2:** The changes of serum TG levels in each time points in rats.

Group	*n*	0 h	2 h	6 h	24 h
CRF	6	152 ± 14	181 ± 26	203 ± 21	213 ± 9 *
saline	6	148 ± 11	151 ± 21	159 ± 16	157 ± 16

Serum TG levels (mg/dL) were measured following intracisternal injection of CRF for 2 h, 6 h and 24 h. Data are expressed as means ± SE; Statistical comparison were made using Student’s *t*-test * *p* = 0.0481 < 0.05.
